# A review on the phytochemical composition and health applications of honey

**DOI:** 10.1016/j.heliyon.2022.e12507

**Published:** 2023-01-11

**Authors:** Gerard-William Zammit Young, Renald Blundell

**Affiliations:** a Department of Physiology and Biochemistry, Faculty of Medicine, University of Malta, Msida MSD2080, Malta; b Centre for Molecular Medicine and Biobanking, University of Malta, MSD2080, Malta

**Keywords:** Honey, Traditional medicine, Modern medicine, Medical grade honey, Antioxidant

## Abstract

**Background:**

Though honey has long been used as medicine, there is a scarcity of knowledge on how it interacts with the body.

**Scope and approach:**

While different types of honey have different chemical and medicinal properties according to their origin, this narrative review seeks to analyse the current knowledge on the chemical composition and therapeutic use of honey. With numerous chemical components, honey has a range of health benefits in multiple disciplines of medicine, and provides an interesting prospect in chemical analysis with regards to identification of its origin.

**Key findings and conclusions:**

There is a great potential for the use of honey in medicine, primarily due to its antioxidant and antimicrobial properties. Recent studies on the phenolic and enzymatic components of honey have made honey's therapeutic method of action in relation to the above properties clearer, still more research needs to be conducted and more innovations need to be tested, for the full potential of honey to be understood.

## Highlights

• Honey, propolis, pollen, and royal jelly, are studied for their role in medicine.
• Different honeys have different chemical and medicinal properties.
• Honey has a wide range of health benefits and has great potential in modern medicine.
• Honey components give benefits, such as antioxidant and antimicrobial properties.

## Graphical abstract

**Figure:**
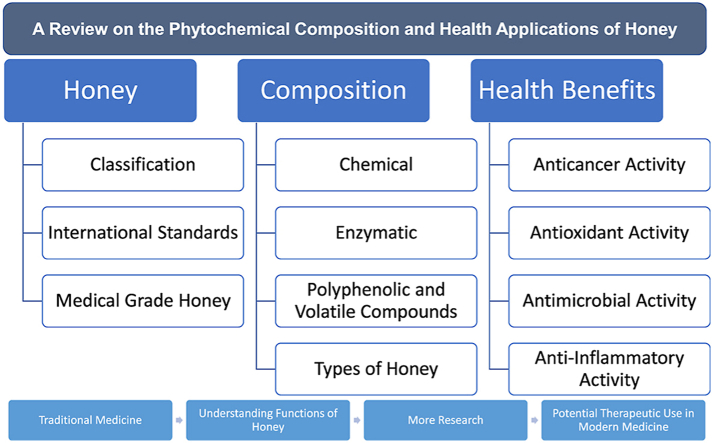

## 1 Introduction

Apitherapy is the use of beehive products for their medicinal and pharmacological properties. It is many times termed an alternative medicine, a last resort to try when everything else fails, yet as more research is done on the subject, more information about its true therapeutic value is being confirmed (Weis et al., 2022).

This narrative review analyses the classification and standards regulating honey, its chemical, phenolic and enzymatic composition, and the various health benefits stemming from the use of honey clinically and in daily life.

While many times it is easy to try to extrapolate the findings of one study to all the types of honey that exist, one would be misguided to do so. The geographical, botanical and even seasonal differences between different honeys leads to different chemical compositions, leading to different therapeutic activities. One therefore needs to research honey from different regions in order to obtain its global properties. In doing so one would also be highlighting and investigating the specific properties and specific types of honey, such as Manuka and Tualang honey (Cebrero et al., 2020).

## 2 International standards and regulations regarding honey

Legislation on what qualifies a substance to be called “honey” emanates from two international standards published by two of the largest international regulatory bodies, that is, the Codex Alimentarius (CA) and the European Council Directive Relating to Honey which are the main legislative standards applicable to most countries around the world. The CA was adopted by the United Nations (UN) via the Food and Agriculture Organization of the UN and the World Health Organisation (WHO) in 1981, revised in 1987 and 2001 and further amended in 2019. The European Directive was adopted in 2001 via the Council of the European Union and amended in 2014 by the European Parliament and Council (Bogdanov et al., 1999). Application of the CA is generally not imposed upon the UN member states, and thus many nations have their own legislation in place, instead of or parallel to the CA. Similarly, some European Union (EU) countries chose to adopt their own forms of regulation (Thrasyvoulou et al., 2018).

### 2.1 Classification of honey

Both standards classify honey as a sweet substance produced by the honeybee, *Apis mellifera*. They both underline the difference between blossom honey or nectar honey and honeydew honey, while the EU legislation also highlights differences between the method of production of different honeys. Furthermore, EU legislation also requires honey to be labelled by country of origin Codex Alimentarius Standard For Honey (2019); Council Directive (2001)/110/EC Relating to Honey (2001); Directive 2014/63/EU of The European Parliament and of the Council Relating to Honey, 2014.

### 2.2 Composition criteria of honey

The two documents have similar but slightly different sets of criteria with regards to the maximum and minimum amounts of the substances making up honey, as explained in Table 1 below (Bogdanov et al., 1999; Codex Alimentarius Standard For Honey, 2019; Council Directive 2001/110/EC Relating to Honey, 2001).

**Table 1:** Composition criteria of honey according to the codex alimentarius and EU regulations.

Criteria	Honey Type	CA	EU
Moisture content	General	≤20%	≤20%
	Heather, Clover	≤23%	≤23%
Sucrose Content	False acacia (*Robinia pseudoacaci*a), leatherwood (*Eucryphia lucida*, *Eucryphia milliganii*), alfalfa (*Medicago sativa*), Banksia (*Banksia menziesii*), Menzies French honeysuckle (*Hedysarum*), Citrus spp., red gum (*Eucalyptus camadulensis*)	≤10%	≤10%
	Lavender	≤15%	≤15%
	Others	≤5%	≤5%
Sum of Glucose and Fructose content	Honeydew honey or blends of honeydew and blossom honey	≥45 g/100 g	≥45 g/100 g
	Others (blossom honey)	≥60 g/100 g	≥60 g/100 g
Water Insoluble Solids content	General	≤0.1 g/100 g	≤0.1 g/100 g
	Pressed Honey	≤0.5 g/100 g	≤0.5 g/100 g
Hydroxymethylfurfural content	General	N/A	≤40 mg/kg
	Tropical Honeys or Tropical Blends	N/A	≤80 mg/kg
Acidity	General	N/A	≤50 meq/kg
	Baker's Honey	N/A	≤80 meq/kg
Diastase Activity (Schade Scale)	General	N/A	≤8
	Honeys with low natural enzyme content and an HMF ≤15 mg/kg	N/A	≤3

### 2.3 Medical grade honey

Medical Grade Honey (MGH) is a type of honey specifically processed to be safe for use in a clinical scenario. This is usually considered sterile and so does not contain any microorganisms. For MGH to be useful, it should be free of toxic material and contaminants and comply with the legal and physicochemical criteria outlined in 2.1 above. Following strict production and storage guidelines organic honey is usually irradiated using gamma radiation to eliminate any pathogenic microorganisms, (Hermanns et al., 2020; Watts and Frehner, 2017).

Honey used to make MGH should not be of commercial origin, but rather organic, extracted from reputable sources. Commercially-available honeys have a lesser degree of antibacterial activity, probably due to thermal treatments, prolonged storage conditions and any possible adulteration which the honey might be subjected to (Bucekova et al., 2020; Hermanns et al., 2020).

It should be noted that in sterilising honey to make MGH, some of its antibacterial properties are lost, with honey samples acquired from beekeepers in Slovakia showing a higher degree of antibacterial activity than MGH (Bucekova et al., 2020). While sterilisation might reduce the functional antimicrobial activity of honey, irradiation is still important in order to eliminate any traces of pathogens which may be present in the honey due to the non-sterile collection and storage processes (Olaitan et al., 2007).

MGH may be used in a variety of ways, including in ointments, gels, or impregnated into wound dressings. They are also used in cases of infection (de Groot et al., 2021; Nolan et al., 2020), wound care (Holubová et al., 2021; Smaropoulos and Cremers, 2020), and burns (Krishnakumar et al., 2020).

## 3 The composition of honey

### 3.1 Chemical composition of honey

Honey is a very complex mixture of various nutrients and components, which vary in percentage concentrations depending on numerous factors. Most honeys only share circa 80% of their physical and chemical composition. Changes in composition could result from geographical and environmental conditions, the floral source that the bee consumes, the type of bee that produces the honey, and the extraction method used. Such variations would lead to different colours, viscosity, taste, and properties of the honey (Ranneh et al., 2021).

Honey is made up of mainly carbohydrates and water, with other substances such as proteins, amino acids, enzymes, polyphenols and other minerals present in much lower quantities. Carbohydrates as a whole represent around 80% of the honey's composition, the bulk of which (75%) is made up of the monosaccharides glucose and fructose. Fructose is usually more abundant than glucose with the exception of a small fraction of honeys such as those coming from *Brassica napus* and *Taraxacum officinale* (Miguel et al., 2017).

There can also be traces of vitamins such as Riboflavin, Pantothenic acid, Niacin, Thiamin, Pyridoxine, and Ascorbic acid as well as minerals such as Potassium, Sulphur, Chlorine, Calcium, Phosphorus, Magnesium, Sodium, Iron, Copper, and Manganese in honey. These can be both from natural sources or from environmental pollutants (Ball, 2007; Bogdanov et al., 2007).

Nectar is the raw material from which the honeybee produces honey, and thus the composition of the nectar from which the honey is produced will greatly affect the composition of the final product. Nectar itself varies greatly in its sugar content, and many bees would prefer nectars with higher sugar content depending on the amount of water availability (Waller, 1972). When nectar is scarce, bees collect the needed nutrients from honeydew produced by smaller insects such as aphids. This type of honey would have a characteristic presence of melezitose. When greater sugar content is present in the collected nectar or honeydew, the resulting honey would have a greater concentration of carbohydrates (Formosa, 2017).

Amino acids can also be detected in concentrations amounting to around 0.5%. They can be found either as free amino acids or as part of proteins. Amino acids such as proline, arginine, glutamic acid, cysteine and aspartic acid can all be detected in honey (Miguel et al., 2017).

Honey adulteration may play a significant role in changing the general composition of honey, especially with regards to its sugar content and physical properties. Generally, adulterated honey contains significantly lower fructose and glucose content and a slight decrease in the glass transition temperature of adulterated honey (Dranca et al., 2022).

Finally, minerals can be detected in varying amounts within honey, as can be seen in Table 2 (Ashagrie Tafere, 2021).

**Table 2:** Minerals found in Honey.

Minerals	Average amount in 100 g honey (mg)
Calcium	4–30
Chlorine	2–20
Copper	0.01–0.1
Iron	1–3.4
Magnesium	0.7–13
Phosphorous	2–60
Potassium	10–470
Sodium	0.6–40
Zinc	0.2–0.5

### 3.2 Enzymes found in honey

Bees utilise the enzymes trypsin, chymotrypsin, elastase, and exopeptidase leucine aminopeptidases, which are found in their midgut, to digest dietary proteins and so be able to produce the proteins and enzymes found in honey (Burgess et al., 1996; Chua et al., 2015).

The vast majority (90%) of proteins found in honey are those belonging to the Major Royal Jelly Protein (MRJP) family, of which there are nine (MRJP1-9). Thus it is quite difficult to detect less abundant low molecular weight proteins found in honey (Rossano et al., 2012). The exact function of all these proteins is not known, but it is thought that they exhibit antioxidant activity (Chua et al., 2015). MRJP1 and MRJP2 have also been shown to have a hypocholesterolemic effect (Chiu et al., 2017), while MRJP3 has been found to have an immunomodulatory effect (Okamoto et al., 2003).

Enzymes related to carbohydrate metabolism, such as diastase, invertase, glucosidase, glucose oxidase, and catalase have been documented as components of honey (Babacan et al., 2002; Pontoh and Low, 2002).

While it is unclear how the enzymes found in honey are made, it is believed that these may either originate from the nectar used by the bee, by microorganisms in the honey, or from the bee itself. Diastase concentration may also be used as an indicator of honey quality, with higher quality honey usually containing more diastase (Ranneh et al., 2021).

In addition to the above-mentioned enzymes, a number of proteolytic enzymes, mostly serine proteases, are also present in honey. These usually originate from the nectar, pollen or from glandular secretions of the bees themselves. Proteolytic enzymes identified in honey mostly correspond to different forms of trypsin and chymotrypsin when compared to their molecular weight and biochemical function (Rossano et al., 2012). Apart from having a digestive function, some of the proteases detected in honey are suspected to have a developmental, defensive or immune function in bees (Paget et al., 2022; Zou et al., 2006). The proteolytic action of these enzymes may explain why honey has much lower amounts of MRJPs when compared with other bee-derived products (Rossano et al., 2012).

### 3.3 Polyphenols and volatile compounds in honey

Phenolic compounds differ greatly from one honey to another, and therefore can be used to determine the origin of honey via high performance liquid chromatography array detection (Miguel et al., 2017). In flowering plants, these compounds may serve as chemical attractants for pollinators such as bees, while in humans these have an impact on the taste and colour of honey (Formosa, 2017).

Polyphenols in honey are mainly flavonoids and phenolic acids and its derivatives, which are thought to give honey its antioxidant and antibacterial properties (Bogdanov et al., 2008). Numerous studies have shown the presence of various phenolic compounds known to have antibacterial properties, as outlined in Table 3.

**Table 3:** A table Linking phenolic and flavonoid compounds found in honey with their antibacterial method of action.

Compound	Molecular Formula	Structure	Mechanism	Reference
Gallic Acid	C_7_H_6_O_5_		Cell membrane disruption and increased pore formation	(Borges et al., 2013; Ranneh et al., 2018)
Ferulic Acid	C_10_H_10_O_4_		Cell membrane disruption and increased cytoplasmic leakage	(Borges et al., 2013; Lima et al., 2022)
Caffeic Acid	C_9_H_8_O_4_		Damage to cell membrane integrity and oxidative stress	(Khan et al., 2021; Ranneh et al., 2018)
Chlorogenic acid	C_16_H_18_O_9_		Increased cell membrane permeability and cytoplasmic and nucleotide leakage	(Cheung et al., 2019; Górniak et al., 2018)
p-Coumaric acid	C_9_H_8_O_3_		Disruption of cell membrane and bacterial DNA binding	(Borges et al., 2013; Ranneh et al., 2018)
Syringic acid	C_9_H_10_O_5_		Dysfunction of cell membrane and inhibition of cellular enzymes	(Ranneh et al., 2018; Srinivasulu et al., 2018)
Vanillic acid	C_8_H_8_O_4_		Disruption of cell membrane and inhibition of biofilm formation	(Cheung et al., 2019; Qian et al., 2020)
Apigenin	C_15_H_10_O_5_		Increase in superoxide production and DNA fragmentation	(Kim et al., 2020; Ranneh et al., 2018)
Catechin	C_15_H_14_O_6_		Production of hydrogen peroxide	(Wu and Brown, 2021; Yayinie et al., 2022)
Luteolin	C_15_H_10_O_6_		Disruption of cell wall and cell membrane, protein expression, and nucleic acid synthesis	(Guo et al., 2020; Tanleque-Alberto et al., 2020)
Pinocembrin	C_15_H_12_O_4_		Increase in cell membrane permeability and disruption of protein and DNA metabolism	(Tanleque-Alberto et al., 2020; Wu et al., 2022)
Galangin	C_15_H_10_O_5_		Inhibition of murein hydrolase gene expression	(Nešović et al., 2020; Ouyang et al., 2018)
Myricetin	C_15_H_10_O_8_		Inhibition of DnaB helicase	(Cheung et al., 2019; Griep et al., 2007)

Phenolic acids and flavonoids can be further subclassified by their structural arrangement and their degree of oxidation. Flavonoids are more abundant than phenolic acids. A study of the digestion and absorption of these compounds would be of great help in understanding the physiology behind the beneficial effect of honey on human health. Still, this is not yet clearly understood. The studies currently available tend to focus more on the flavonoid component of honey. Flavonoids are known to be hydrolysed in the intestine and transported into the epithelium via sodium-dependent glucose transporter 1, where they may have an inhibitory action on Na-dependent facilitated diffusion of monosaccharides into the cells from the intestinal lumen (Cianciosi et al., 2018).

Compounds such as acids, alcohols, aldehydes, ketones, terpenes, norisoprenoids, benzene compounds and their derivatives, furan and pyran derivatives and other hydrocarbons all contribute as volatile compounds within honey. Similar to polyphenols, the fraction of these compounds which come from floral origins may be used to determine the botanical and geographical origin of the honey. Furthermore, the variation of these volatile compounds contribute to the aromas of different honeys (Manyi-Loh et al., 2011).

### 3.4 Different types of honey

In this section some different types of honey according to their visual appearance as well as their floral origins will be listed and discussed. These should not be taken as fully conclusive, as honey is affected by other factors such as weather and environmental conditions. Mixtures of honeys of various floral origins may also not follow Table 4 (Collins, 2010; National Honey Board, n.d.).

**Table 4:** A table comparing various types of honey with their country of origin, colours and flavours.

Name	Country of Origin	Colour and Flavour
**Dark Honeys**		
Avocado	USA, especially California; Mexico; Australia	Dark amber colour; rich flavour of caramelised molasses
Buckwheat	USA; Canada; China; Russia	Very dark brown colour; strong molasses and malt flavour
Chestnut	Southern Europe	Dark amber colour; sharp, bitter flavour
Eucalyptus	USA, especially California; Australia; New Zealand; Italy	Dark amber colour; caramel flavour
Hawthorn	North America; New Zealand; Western and Northern Europe	Dark amber colour; nutty flavour
Heather	Europe, especially UK, Ireland and Scnadinavia	Dark amber or reddish brown colour; bitter-sweet taste
Honeydew	Europe, especially Germany, Greece and Turkey; New Zealand	Dark amber colour; strong rich flavour
Manuka	New Zealand	Dark colour; herbaceous taste
Rewarewa	New Zealand	Dark amber colour; rich malty flavour
Tulip Tree	Eastern USA	Very dark amber colour; strong flavour
**Medium Honeys**		
Blackberry	UK; Canada	Light chestnut colour; coarse flavour
Coconut Palm	USA; West Indies	Amber colour; strong flavour
Dandelion	Worldwide	Golden yellow colour; strong flowery taste
Lavender	Europe, especially France and Spain	Golden colour; flowery taste
Lime Tree	Europe; Canada; USA	Amber or light yellowish green colour; strong flavour with vanilla taste
Mixed Meadow Flowers	Worldwide	Golden yellow; full flavour
Orange Blossom	USA; New Zealand; Asia	Medium amber colour; fruity taste
Rosemary	Mediterranean Europe	White to reddish gold colour; medium flavour
Thyme	Mediterranean Europe; North America; New Zealand	Bright amber colour; intense aromatic flavour
Tupelo	Southeastern USA	Light amber to medium yellow colour; sweet flavour
**Light Honeys**		
Acacia	USA; Europe	Pale golden yellow colour; sweet, delicate flavour
Alfalfa	USA, especially California; Canada	Light amber or pale white colour; mild minty flavour
Apple	Worldwide	Light amber colour; good flavour with apple hints
Blueberry/Cranberry	USA; Canada; Europe	Light amber colour; full fruity flavour
Borage	UK; Canada; New Zealand	Pale yellow or white; light flavour
Clover	USA; Canada; Egypt; Europe; Australia; New Zealand	Pale amber, yellow or white colour; flowery flavour
Cotton	Egypt; Southern USA	Pale amber colour; light flavour
False Acacia	Europe; North America	Light colour; sweet flavour
Fireweed	Worldwide	Pale amber to white colour; subtle tea flavour
Fuchsia	Europe; New Zealand; North and South America	Light colour; mild flavour
Goldenrod	Europe; North America	Light-medium gold colour; slightly strong spicy flavour
Holly	Southern USA; Western and Southern Europe	Pale colour; fine flavour
Ivy	Europe; Asia; North America	Grey-white or yellow colour; bitter flavour
Knapweed	Ireland	Light amber colour; mild tangy flavour
Leatherwood	Tasmania	Light golden yellow colour; strong spicy taste
Maple	Canada; USA; UK	Pale yellow sometimes greenish colour; mild taste
Melilot	Worldwide	Pale greenish-yellow colour; slight cinnamon flavour
Mesquite	Mexico and USA	Light colour; smoky molasses flavour
Oil-seed rape	USA; Europe; Asia	White to light amber colour; mild taste
Rata	New Zealand	Water white colour, medium-bodied flavour
Sainfoin	North America; Europe	Lemon yellow colour; aromatic flavour
Sunflower	North America; Europe; Russia; China	Yellow colour; light flavour

## 4 The health benefits of honey

### 4.1 The anticancer activity of honey

Cancer treatment generally involves drugs which induce apoptosis in the cancerous cells. Honey has a similar effect in depolarising the mitochondrial membrane and stimulating expression of caspase 3 and 9 in cancer cells, thus inducing apoptosis (Fauzi et al., 2011).

**Figure 1:**
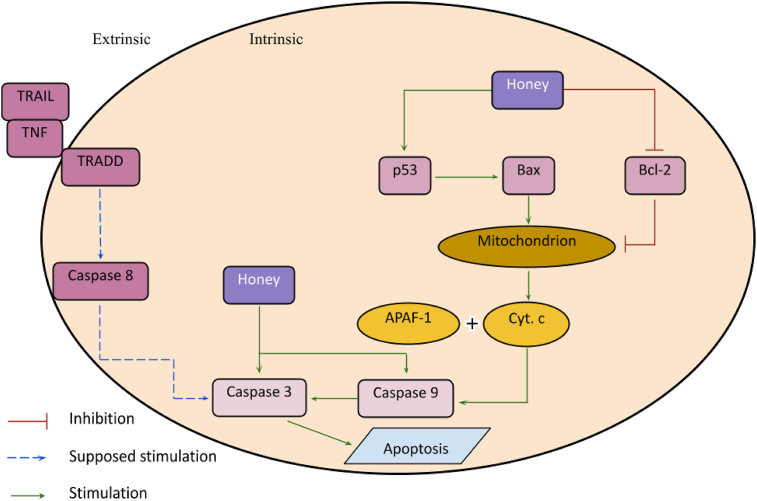
The effect of honey on the apoptotic pathway.

Honey also stimulates p53 expression, while down-regulating B cell lymphoma 2 protein (Bcl-2), which are proapoptotic and antiapoptotic proteins respectively. p53 thus depolarises the mitochondrial membrane via Bcl-2-associated X protein while Bcl-2 stops maintaining the resting potential of the mitochondrial membrane. The membrane depolarisation induces activation of cytochrome C, which in the presence of apoptotic protease activating factor 1 stimulates caspase 9 expression and so upregulates the apoptotic pathway. This thus induces apoptosis of cancerous cells. This process is explained in Figure 1 below (Ahmed and Othman, 2013).

Furthermore, honey has been shown to decrease nuclear protein Ki-67 presence when administered orally with *Aloe vera*. This protein is present in the cell proliferation phases (G1, S, G2 and mitosis) but absent during the resting phase (G0). The decrease in Ki-67 was noted during all phases of cell replication, thus decreasing the rate by which cancerous cells divide, and so decreasing tumour growth (Tomasin and Gomes-Marcondes, 2011).

Another theory is that honey prevents cancer due to its antimutagenic activity against physical and chemical mutagens. Honey suppresses the error-prone repair pathway in bacterial cells and thus decrease mutations in these bacteria when dividing (Saxena et al., 2012).

Proteins in honey have been shown to induce the release of tumour necrosis factor α (TNFα) by macrophages (Majtán et al., 2006; Simúth et al., 2004). This increase in TNFα induces the release of reactive oxygen species (ROS) and induces the immunologic response to destroy the cancerous cells (Chan-Zapata and Segura-Campos, 2021; MacEwan, 2002).

### 4.2 The antioxidant activity of honey

The oxidation of molecules, which is crucial for the healthy operation of the cell, is prevented by antioxidants. Oxidation would successively harm tissues, organs and hence, the physiological functioning of the organism. Antioxidants are maintained in balance by a sophisticated system within the body. Food containing antioxidants has been demonstrated to help control this system and so promote health. While not yet confirmed through clinical trials, *in vivo* studies have shown that honey alleviates oxidative stress in various organ systems (Meo et al., 2017).

Honey contains a number of molecules, such as flavonoids, glucose oxidase, catalase, phenolic acids, ascorbic acid, and carotenoids which have been shown to have antioxidant activity both *in vitro* and *in vivo*. Many of these compounds exhibit a combined synergistic effect, and hence, honey is regarded as a natural antioxidant (Bogdanov et al., 2008).

While the handling and processing of honey may impact the antioxidant activity it exhibits, the most important factor affecting its antioxidant capacity is the geographical and botanical origin of the honey itself. This is correlated with the total phenolic content of honey, with honeys containing higher amounts of phenolic acids exhibiting higher antioxidant activity. As darker honeys tend to contain more phenolic acids, colour may be associated with antioxidant activity (Eteraf-Oskouei and Najafi, 2013).

While classical examples of antioxidants such as Vitamins C and E become pro-oxidants themselves when in large doses, honey has been shown not to exhibit this behaviour when given in larger amounts. This is thought to be due to the presence of more than one compound having antioxidant activity within honey, which helps to reconvert any compounds which become pro-oxidants into their active, antioxidant forms (Erejuwa et al., 2012).

The antioxidant activity of honey has been correlated with the prevention of several disorders such as cardiovascular diseases (Rakha et al., 2008), diabetes (Erejuwa et al., 2010) and cancer (Hassan et al., 2012).

### 4.3 The antimicrobial activity of honey

Honey has a number of properties which make it an ideal antimicrobial agent. The high sugar content, low pH, hydrogen peroxide, polyphenol compounds, and antimicrobial peptides all contribute in fighting against various types of pathogenic organisms, and further down the line, tissue repair (Almasaudi, 2021).

Throughout history, honey has been used as an antibacterial agent. Recent research has shown that honey does actually have antibacterial effects on aerobic, anaerobic, Gram-positive and Gram-negative bacteria, while it may also contain spores introduced during the production process (Olaitan et al., 2007).

A potential use of honey in modern medicine is in the treatment of patients presenting with Methicillin-resistant *Staphylococcus aureus* (MRSA) (Ayefoumi Adinortey et al., 2022). Numerous studies have shown positive results in the susceptibility of MRSA to honey. This provides a potential for a route of alternative treatment for antibiotic resistant bacteria, as well as reducing the number of antibiotics used during treatment, which may themselves be a cause to further antibiotic resistances (Chambers, 2006; Maeda et al., 2008; Natarajan et al., 2001).

Honey has also been found to have antifungal properties. *In vitro* studies have concluded that honey has an effect on *Candida* (de Groot et al., 2021) and *Rhodotorula* (Ahmed et al., 2013), which are opportunistic pathogenic yeasts. While no information on the clinical use of honey to treat fungal infections could be found at the time of writing, researchers are hopeful that the antifungal property of honey may be used in the near future for the treatment of antifungal resistant strains (Irish et al., 2006; Moussa et al., 2012).

Further to the antibacterial and antifungal properties exhibited by honey, it has been found to show a degree of antiviral activity as well. The antiviral activity of honey has been best shown in the treatment of skin lesions caused by the herpes simplex virus. In this regard, honey has shown faster treatment time than that taken by the normally prescribed antiviral acyclovir (Al-Waili, 2004; Rocha et al., 2022).

Despite antimicrobial resistance being constantly on the rise worldwide, there has never been a case of microbial resistance to honey reported, leading to honey frequently being used as a last resort. This is possibly due to honey having a number of different components which may exhibit antimicrobial properties (Almasaudi, 2021; Mandal and Mandal, 2011).

### 4.4 The anti-inflammatory activity of honey

Honey contains various compounds which have anti-inflammatory potential. The most notable anti-inflammatory effect exhibited by a component of honey is that of flavonoids, which mediates various cytokines such as TNF-α, interleukin (IL)-2, IL-10, IL-12p70, nitric oxide and interferon‐gamma (Silva et al., 2021, Silva et al., 2020).

Honey has been linked to its anti-inflammatory properties through *in vitro* studies (Candiracci et al., 2012), in animal studies (Leong et al., 2012) and also in clinical trials (Al-Waili and Boni, 2003; Subrahmanyam, 1998). With further research and clinical trials on this effect of honey, new drugs may be produced from this natural product. They may have all the necessary anti-inflammatory properties needed while also reducing or eliminating altogether the side effects associated with mainstream drugs used to treat chronic inflammation such as corticosteroids and non-steroidal anti-inflammatory drugs (Eteraf-Oskouei and Najafi, 2013).

Honey is attributed with the inhibition of the formation of ROS and the suppression of cyclooxygenase-2 enzymes, both of which are related to the pro-inflammatory pathway. This may be due to the mediation of the above-mentioned cytokines or due to gene suppression by transcription factors triggered by honey. Through this activity, honey thus exhibits an inhibitory effect on chronic inflammation (Ahmed and Othman, 2013; Leong et al., 2012; Ranneh et al., 2021).

## 5 Conclusion and discussion

Since the beginning of civilisation many claims were aimed at honey and its therapeutic use. Even today, a lot of research is being done to prove or disprove claims made in traditional medicine about the action of honey and hive products. While *in vitro* and *in vivo* studies provide valuable insight into the potential therapeutic benefits of honey, it would be misguided to draw the conclusion that these effects hold true in the human body. More research and most importantly more clinical trials are needed to evaluate the use of honey and other products of the beehive in medicine. There is still no clear consensus on the practice of apitherapy between regions of the world, and as such one should highlight the need to standardise and expand the present knowledge on apitherapy through collaborative research between chemists, medics and professionals within the industry.

Other issues which still need to be addressed are the standardisation of MGH components, the types of honey used to make MGH, and the process used to sterilise honey of any potentially pathogenic components while sparing its beneficial properties.

Furthermore, adulteration leads to drastic changes in honey content, which in turn may adversely affect body organ systems, especially the renal system (Fakhlaei et al., 2020).

As can be seen throughout this article, a lot of potential exists surrounding apitherapy, especially with regards to the treatment of multidrug resistant infections with honey. Although there have been significant advances in recent years in understanding the function and physiology of apitherapy, there is still a need to explore further and build upon this knowledge, as evidenced by a large number of articles that were referred to which highlighted the need for further research on the subject. In addition to this, contrary to other products such as pollen and bee venom, honey allergies are extremely uncommon. While these occur very rarely, care should be taken to make sure that there is no adverse reaction when using honey medically, as symptoms may range from simple to very severe (Altameemi et al., 2022).

It should be noted that effectively, the intake of honey has little to no side effects, and it is only in rare cases that humans have exhibited signs and symptoms of honey allergies (Altameemi et al., 2022).

## Declarations

### Author contribution statement

All authors listed have significantly contributed to the development and the writing of this article.

### Funding statement

This research did not receive any specific grant from funding agencies in the public, commercial, or not-for-profit sectors.

### Data availability statement

Data included in article/supp. material/referenced in article.

### Declaration of interests statement

The authors declare no competing interests.

### Additional information

No additional information is available for this paper.
